# Astrocytes underlie a faster-onset antidepressant effect of hypidone hydrochloride (YL-0919)

**DOI:** 10.3389/fphar.2023.1175938

**Published:** 2023-03-29

**Authors:** Jin-Feng Li, Wen-Yu Hu, Hai-Xia Chang, Jin-Hao Bao, Xiang-Xi Kong, Hui Ma, Yun-Feng Li

**Affiliations:** ^1^ Beijing Institute of Basic Medical Sciences, Beijing, China; ^2^ Institute of Neuroscience, Hengyang Medical College, University of South China, Hengyang, China; ^3^ College of Pharmacy, Shandong First Medical University & Shandong Academy of Medical Sciences, Taian, China; ^4^ Jiangsu Province Key Laboratory of Anesthesiology, Jiangsu Province Key Laboratory of Anesthesia and Analgesia Application, NMPA Key Laboratory for Research and Evaluation of Narcotic and Psychotropic Drugs, Xuzhou Medical University, Xuzhou, China; ^5^ State Key Laboratory of Toxicology and Medical Countermeasures, Beijing Key Laboratory of Neuropsychopharmacology, Beijing Institute of Pharmacology and Toxicology, Beijing, China

**Keywords:** depression, astrocytes, YL-0919, RNA-seq, mitochondria

## Abstract

**Introduction:** Major depression disorder (MDD) is a common and potentially life-threatening mental illness; however, data on its pathogenesis and effective therapeutic measures are lacking. Pathological changes in astrocytes play a pivotal role in MDD. While hypidone hydrochloride (YL-0919), an independently developed antidepressant, has shown rapid action with low side effects, its underlying astrocyte-specific mechanisms remain unclear.

**Methods:** In our study, mice were exposed to chronic restraint stress (CRS) for 14 days or concomitantly administered YL-0919/fluoxetine. Behavioral tests were applied to evaluate the depression model; immunofluorescence and immunohistochemistry staining were used to explore morphological changes in astrocytes; astrocyte-specific RNA sequencing (RNA-Seq) analysis was performed to capture transcriptome wide alterations; and ATP and oxygen consumption rate (OCR) levels of primary astrocytes were measured, followed by YL-0919 incubation to appraise the alteration of energy metabolism and mitochondrial oxidative phosphorylation (OXPHOS).

**Results:** YL-0919 alleviated CRS-induced depressive-like behaviors faster than fluoxetine and attenuated the number and morphologic deficits in the astrocytes of depressed mice. The changes of gene expression profile in astrocytes after CRS were partially reversed by YL-0919. Moreover, YL-0919 improved astrocyte energy metabolism and mitochondrial OXPHOS in astrocytes.

**Conclusion:** Our results provide evidence that YL-0919 exerted a faster-onset antidepressant effect on CRS-mice possibly *via* astrocyte structural remodeling and mitochondria functional restoration.

## Introduction

Major depressive disorder (MDD) is a pervasive and devastating mental illness with heavy social and economic burdens and is characterized by anhedonia, low motivation, cognitive and memory impairment, high suicide rate, etc. (Malhi and Mann, 2018). During the COVID pandemic, the prevalence of MDD has grown dramatically even among youths (Collaborators, 2021; Thapar et al., 2022). While more than 350 million people are currently affected by MDD, few novel antidepressants are available with low side effects, rapid onset, and sustained action (Oslin et al., 2022).

Astrocytes, a type of glia, are the most abundant cell population in the central nervous system (CNS) and play a critical role in maintaining brain homeostasis (Kofuji and Araque, 2021). In addition to basic support and protection, astrocytes also participate in neuronal energy supply, neurotransmitter recycling (Stobart et al., 2018), ion homeostasis regulation, and synaptic transmission (Kruyer et al., 2023), as well as neuroimmunomodulation and other roles (Diaz-Castro et al., 2021). Therefore, their pathological deficits are closely related to many CNS diseases (Lee et al., 2022). The classical theory holds that antidepressants work by enhancing neuronal plasticity and regulating neural circuit function. In recent years, cumulative evidence has indicated that structural and functional abnormalities in astrocytes are key mechanisms leading to MDD (Zhou et al., 2019). Notably, astrocytes generate energy through mitochondrial oxidative metabolism and “intelligently recharging” the neurons according to their activity; thus, the mitochondria may determine astrocyte functional regulation in depression. However, the specific mechanism remains unclear.

Hypidone hydrochloride (YL-0919) is a novel antidepressant in phase II clinical trials that was developed by our laboratory. Previous studies demonstrated its significant rapid antidepressant effects in a variety of animal depression-like models (Zhang et al., 2017; Ran Y. H. et al., 2018; Yin et al., 2020; Zhang et al., 2022). Furthermore, YL-0919 also promoted cognition and learning (Chen et al., 2018). However, the underlying astrocyte-specific mechanisms require further exploration.

In the present study, we first validated the faster antidepressant activity of YL-0919 compared to fluoxetine, which usually shows effects after 3–5 weeks in a chronic stress depression model. Then, we explored the effects of YL-0919 on astrocytes in the medial prefrontal cortex (mPFC) and hippocampus (HIP), which are critical for emotion regulation. Moreover, cell-specific and high-throughput exploration by RNA-Seq showed that YL-0919 partially reversed CRS-induced expression profile alterations in astrocytes. Finally, we found that mitochondrial function may be the key to a new astrocyte-specific mechanism by which YL-0919 shows antidepressant effects. In summary, our findings shed light on the mechanisms underlying the faster-onset antidepressant effect of YL-0919 and advance our understanding of the complex astrocytic pathogenesis of depression.

## Methods and materials

### Animals

Male C57BL/6 mice (7 weeks old) were purchased from Beijing SPF Biotechnology (Beijing, China). The animals were housed 4–5 per cage under controlled temperature and kept on a 12-h light/dark cycle (lights on between 07:00 and 19:00), with *ad libitum* access to food and water. Initially, the mice were acclimated for 1 week to reduce stress and undergo the sucrose preference baseline test. All experimental animal procedures were approved by the Institutional Animal Care and Use Committee of the Beijing Institute of Basic Medical Sciences. All surgical procedures were performed under anesthesia, and every effort was made to minimize suffering.

### Chronic restraint stress procedure

The CRS procedure was performed as previously described (Du Preez et al., 2021). CRS mice were individually placed into a modified well-ventilated 50 mL centrifuge tube (NEST Biotechnology) daily from 10 a.m. to 16 p.m. for 14 days. The mice could change position from supine to prone but were not able to move forward or backward in the tubes. The control mice remained in their home cages and were allowed to move freely. After restraint stress, the mice were released from the tube and returned to their cages.

### Pharmacology administration

YL-0919 (white powder, purity ≥99.8%, #D5222-18-001) was purchased from Zhejiang Huahai Pharmaceutical Co., Ltd. Fluoxetine was purchased from Sigma-Aldrich (#56296-78-7). Both YL-0919 (2.5 mg/kg) and fluoxetine (5 mg/kg) were dissolved in sterile physiological saline, as previously described (Manosso et al., 2013; Qin, 2014; Li, 2020). The mice were administered the drugs *via* intragastric gavage (i.g.) at a volume of 10 mL/kg at the same time of the day, 1 h before restraint, and continuously until euthanasia.

### Behavioral assays

**FIGURE 1:**
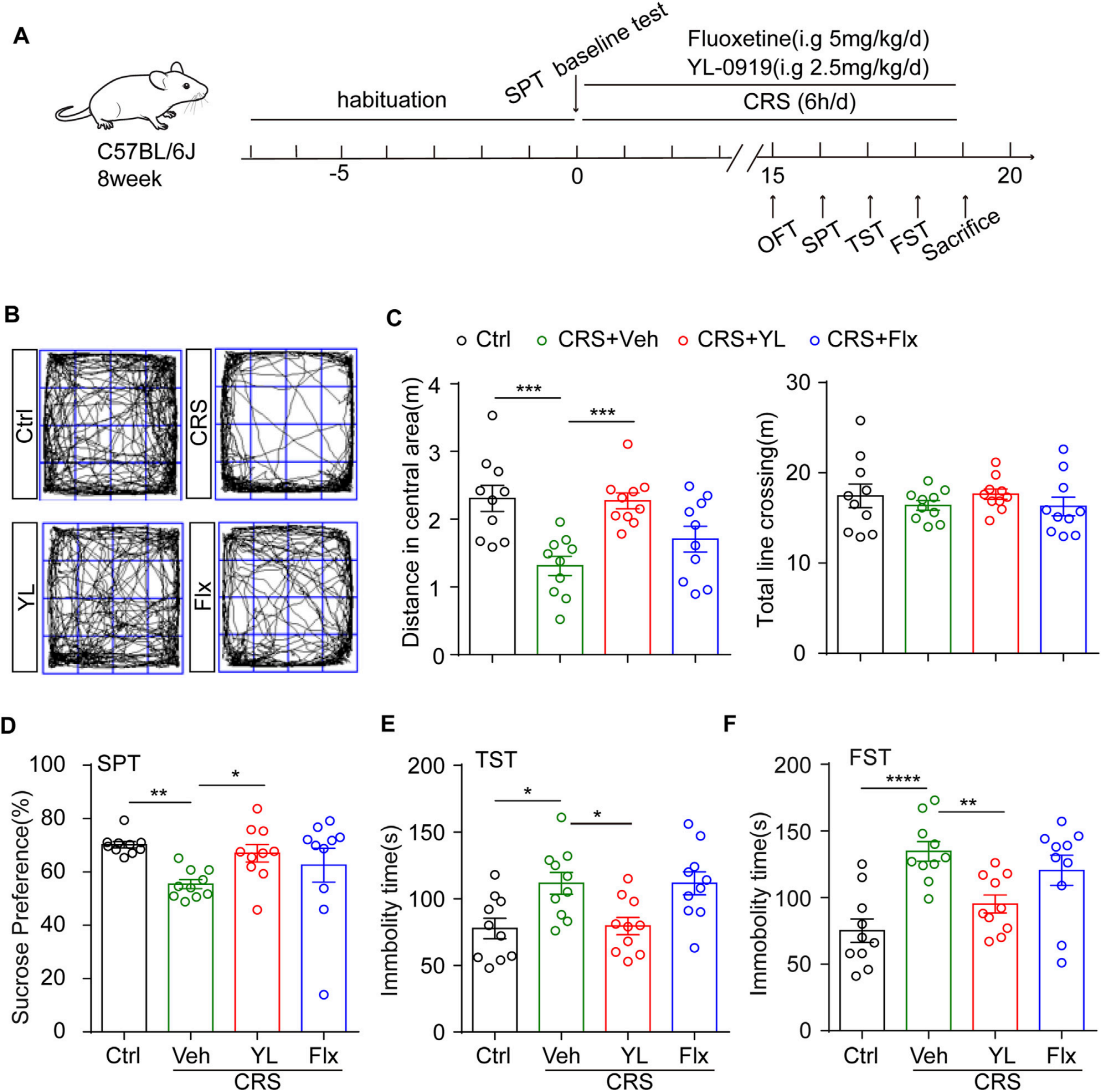
YL-0919 concomitant treatment alleviates depression-like behaviors induced by CRS. **(A)** Experimental procedure. Timeline of mice adaption, CRS exposure, drug administration strategy, and behavioral tests. **(B)** Representative trace recordings of the OFT. Quantification of the distance traveled in the central zone **(C, left)** (F (3, 36) = 8.569, *p* = 0.0002) and the total distance moved in the OFT **(C, right)** (F (3, 36) = 0.5748, *p* = 0.6353). **(D)** Percentage of 1% sucrose consumption in the SPT after CRS exposure and drug treatment (F (3, 27) = 3.14, *p* = 0.0416). **(E,F)** Quantification of the time spent immobile in the TST (F (3, 36) = 5.918, *p* = 0.0022) and FST (F (3, 36) = 9.143, *p* = 0.0001). Data are expressed as mean ± SEM, *n* = 10 in each group. For SPT, one-way ANOVA, followed by Fisher’s LSD, was used; otherwise, Bonferroni’s multiple comparisons test was applied. *p < 0.05; **p < 0.01; ***p < 0.001; ****p < 0.0001.

The behavioral assays described in the following paragraphs are in the order of assay performance. After 14 days of treatment, the mice were tested for behavior in the order from less to more stressful tests, as indicated in Figure 1A, with a 24 h interval between tests to permit ample resting of the mice and prevent interference between tests.

### Sucrose preference test

The sucrose preference test (SPT) was performed, as previously described with minor modifications (Liu et al., 2018). The mice were housed singly and habituated with two bottles of 1% (w/v) sucrose solution for 1 day before being exposed to one bottle of 1% (w/v) sucrose solution and one bottle of drinking water for 12 h in the dark phase. The bottle positions were switched after 6 h to avoid place preference. The total consumption of each fluid was measured by micro-balance, and sucrose preference was defined as the average sucrose consumption ratio (consumption of sucrose/consumption of total liquid × 100%) during the 12-h test.

### Open field test

The open field test (OFT) was performed as previously described with minor modifications (Shi et al., 2021). The open field apparatus consisted of four 50 cm × 50 cm × 20 cm opaque white arenas. The intensity of the ambient light in the OFT was 200 ± 0.5 LUX. The mice were acclimated to the experimental test room for at least 60 min before testing. To start a session, a mouse was placed in the center of the arena and allowed to freely explore for 15 min with video recording. The total distance traveled (m), time (s) spent in the center zone (25 cm × 25 cm), and center entries were automatically quantified by software (ANY-maze, Stoelting, United States), and the last 10 min were used for statistical analysis.

### Tail suspension test

The tail suspension test (TST) was performed as previously described with minor modifications (Liu et al., 2007). Mice were suspended individually with secured tails by adhesive tape and dovetail clip to the ceiling of a shelf 40 cm above the floor. Each mouse was suspended and recorded for 6 min, and the immobility time for the last 4 min was measured. Immobility was defined as the time that the mice spent suspended with all limbs motionless.

### Forced swimming test

The forced swimming test (FST) was performed as described previously (Armario, 2021). In brief, each mouse was placed for 6 min in a glass cylinder (height: 25 cm; diameter: 18.5 cm) filled with water (24°C ± 1°C) to a depth of 15 cm. The water depth was adjusted so that the animals were forced to swim or float without their hind limbs touching the bottom. The duration of immobility was measured for the last 4 min by averaging the results from two researchers. Immobility was defined as the time during which mice floated with all limbs motionless or just made the minimal movements necessary to keep their heads above water. The mice were gently dried after removal from the water and returned to their cages.

### Immunofluorescence and immunohistochemical staining

The mice were euthanized with tribromoethanol and then intracardially perfused with ice-cold saline. The brains were fixed with 4% paraformaldehyde (PFA) for at least 48 h and processed for immunochemistry. Coronal sections (30 μm) of the mouse brains were cut using a cryostat (Leica, # CM3050S) and stored at −20°C in cryoprotective storage solution (30% sucrose, 1% polyvinyl-pyrrolidone, 5 mM Na2HPO4, 20 mM NaH2PO4, and 30% ethylene glycol) until use. Upon use, the sections were washed three times with PBS. For GFAP staining, the sections were first subjected to heat-induced antigen retrieval in a 50 mM sodium citrate solution containing 0.05% Tween-20 for 30 min at 95°C. After natural cooling to room temperature, the brain sections were washed three times with PBS and incubated with blocking buffer (0.3% Triton-X-100, 5% goat serum, and 2% BSA in PBS) for 1.5 h at room temperature, followed by incubation with primary antibody (mouse monoclonal anti-GFAP, Millipore, #MAB360) overnight at 4°C. On the second day, the sections were incubated with an Alexa Fluor 488 or TRITC conjugated isotype-specific secondary antibody for 1 h at room temperature. Images were then acquired using a Nikon confocal microscope. Immunohistochemical staining was carried out using an SAP kit (ZSGB BIO, #SAP-9100) and visualized using a DAB kit (ZSGB BIO, #ZLI-9018) after reaction with 30% hydrogen peroxide. The images were then scanned using a Pannoramic Scan Ⅱ device (3DHISTECH) and captured using CaseViewer.

### Cell density measurement

In each image, regions of interest (ROIs) were defined using free-hand drawing in Image-Pro. The areas of each ROI were measured. For S100β^+^ or GFAP^+^ cell counting, at least half of the cell nucleus was visible on the edge of the ROI, and the cells included in the analysis were not adhered to blood vessels.

### Morphological analysis

Morphological analysis was performed using the Ghosh Lab Sholl analysis plugin in ImageJ. In brief, the maximum intensity projection of confocal images containing GFAP-stained astrocytes was generated and saved in TIFF format. The 2-D images were then binarized in ImageJ. Next, we used the “analyze particles” tool to eliminate the background using size exclusion. The centroid for Sholl analysis was selected based on the morphology of the original GFAP-stained images. The Ghosh Lab Sholl analysis plugin was then used to generate Sholl analysis vectors. A 15-period moving average was calculated for every cell to generate smooth curves for the Sholl analysis. Approximately 30 cells (from three sections per mouse) from three to four mice per group were used for Sholl analysis per region per group. The results were represented as mean ± SEM of all cells.

### Astrocyte isolation

The mice were deeply anesthetized with pentobarbital sodium and perfused transcardially with ice-cold saline. Whole brains without the olfactory bulb and cerebellum were immediately removed and washed with 4°C prechilled sterile phosphate-buffered saline. The brain tissue was mechanically and enzymatically dissociated using the Adult Brain Dissociation Kit (Miltenyi Biotec, #130-107-677) according to the manufacturer’s instructions. After myelin was removed using the cell debris removal buffers, the total cell pellet was resuspended in PBS containing 0.5% BSA, followed by the isolation of specific cell types. Briefly, astrocytes were isolated using anti-ACSA-2 MicroBeads (Miltenyi Biotec, #130-097-678) on a MACS^®^ MultiStand Separator, according to the manufacturer’s instruction. For RNA extraction, isolated cells were frozen using liquid nitrogen and stored at −80°C before use. Isolated cells from two mice were pooled for each sample.

### RNA-sequencing (RNA-seq) analysis

RNA-sequencing analysis was completed by LC-Bio Technologies (Hangzhou, China, order number 2022B24wyC00). Briefly, the total RNA of primary mouse astrocytes was collected in TRIzol reagent (Thermofisher, #15596018) with three biological replicates for each group. A total of 5 μg of total RNA was used for RNA-seq library construction. The 2 × 150bp paired-end sequencing (PE150) was performed on an Illumina Novaseq™ 6000 system, following the vendor’s recommended protocol. MI technology was used to label each sequence fragment with sequence tags, which minimized the interference of duplication generated by PCR amplification on the quantitative accuracy of the transcriptome. The RNA-seq reads of all samples were aligned to the mouse genome (GRCh37/hg19) using the Hisat2 (2.2.1) package. Transcript abundance was quantified as fragments per kilobase of transcript per million mapped reads (FPKM) value for mRNA expression levels. The threshold of significantly differential expression was set at *p* < 0.05 and |log2 (fold change)| ≥ 1. We performed gene set enrichment analysis (GSEA) using GSEA (v4.1.0) and MSigDB software to identify whether a set of genes showed significant differences between groups in specific GO terms and KEGG pathways. GO terms, KEGG pathways meeting this condition with |NES|>1, NOM *p* < 0.05, and FDR<0.25 were considered to be different between two groups. The RNA-Seq data presented in the study are deposited in the SRA repository, accession number PRJNA944268.

### Primary astrocyte culture

Primary astrocytes were generated from newborn C57BL/6 mice within 24 h. The procedure was modified from a previous report (Pan et al., 2022). Briefly, brains dissected were taken after disinfection with 75% alcohol and then transferred to a Petri dish containing precooled PBS to clean the blood cells. The meninges and blood vessels on the brain surface were stripped under the microscope. Next, 0.25% trypsin was added for digestion at 37°C for 10 min and stopped with complete medium DMEM-F12 (Invitrogen) with 10% fetal bovine serum (FBS, Gibco) and 1% penicillin-streptomycin (Invitrogen). After the brain tissue was fully dissociated and no small clumps were observed, the liquid was passed through a 70-μm nylon cell strainer. The filtrate was then centrifuged at 1500 rpm for 10 min, and the bottom cells were resuspended in medium. After thoroughly blowing, the cells were inoculated into the T25 culture flasks coated with poly-D-lysine and placed in an incubator at 37°C and 5% CO_2_ for culture. The liquid was changed every 2 days. After 10 days of culture, primary microglia were separated with a slight bump and the astrocytes were collected for the experiments.

### Quantitative PCR

Total RNA was extracted from experimental cells using TRIzol™ reagent (Invitrogen, #15596018), as previously described (Peng et al., 2021). Total purified RNA (1 μg) was used for the reverse transcriptase reaction using a cDNA synthesis kit (Monad, #MR05001M) according to the manufacturer’s instructions. Quantitative real-time PCR (qRT-PCR) was performed using SYBR Green (GenStar #A304-10) on a Bio-Rad iCycler iQ Real-Time PCR system (Thermo Fisher Scientific). β-Actin was used as the optimal internal standard control. The mRNA expression analysis was performed using the -ΔΔCT method. The primer sequences for qPCR are listed in Supplementary Table S1.

### Cell viability assay

The cytotoxic effects of the indicated compounds on experimental cells were determined using Cell Counting Kit-8 (CCK-8) (TransGen Biotech, #FC101-02), according to the manufacturer’s protocol. Briefly, 5×10^3^ cells per well were plated into 96-well plates the night before and then treated with drugs at the indicated doses for 24 h. Then, 10 μL of CCK-8 solution was added at the indicated time and the plates were incubated for 2 h in a cell culture incubator. The absorbance at 450 nm was measured using a microplate reader (TECAN M200, Switzerland).

### Measurement of ATP levels

ATP levels were determined using CellTiter-Glo reagent (Promega, #G7573) according to the manufacturer’s protocol. In brief, primary astrocytes were plated onto 96-well plates and cultured overnight. The following day, after the drug treatments, 50 μL of supernatant was transferred to another 96-well plate for the measurement of extracellular ATP level and the remaining liquid was discarded. For intracellular ATP level measurement, 100 μL of lysis buffer containing luciferase reagents was added to the cells and incubated for 10 min at room temperature. Fluorescence intensity was determined using a microplate reader, and the data were normalized to protein concentration.

### Seahorse extracellular flux assay

The oxygen consumption rate (OCR) was determined based on mitochondria oxidative phosphorylation (OXPHOS) using a Seahorse XF Cell Mito Stress Test Kit (Agilent Technologies, #103015-100) and measured on a Seahorse XFe96 Analyzer, following the manufacturer’s instructions and modified according to previous studies (Yao et al., 2020). In brief, primary astrocytes were seeded onto a Seahorse XF 96-well culture microplate overnight. The cells were then treated with drugs after achieving adherence. Upon measurement, the cells were washed twice with XF assay medium (Agilent Technologies, #102353-100) and maintained in this medium. After the baseline measurements, oligomycin (Oligo; 2 μM), p-trifluoromethoxy carbonyl cyanide phenylhydrazone (FCCP; 1 μM), and rotenone/antimycin A (0.5 μM) were injected into the wells sequentially at specific time points for OCR analysis. Seahorse XF 96 Wave software was used to analyze the data. The results were normalized to protein concentration, and the data are presented as pmol/min.

### Statistical analysis

All data are expressed as means ± SEM. GraphPad Prism version 6.0 was used for statistical analysis. The significance of differences was calculated using a two-tailed Student’s t*-*test or one-way ANOVA or two-way ANOVA. Fisher’s LSD or Bonferroni’s multiple comparisons test or Tukey’s *post hoc* test was performed for comparisons among groups. All n represent biologically independent experiments. For all tests, *p* < 0.05 was considered statistically significant (∗*p* < 0.05, ∗∗*p* < 0.01, ∗∗∗*p* < 0.001, and ∗∗∗∗*p* < 0.0001).

## Results

### YL-0919 has a faster-onset antidepressant effect in CRS mice

CRS is a widely used animal model of depression (Wang et al., 2017; Zheng et al., 2022); hence, we used the CRS procedure shown in Figure 1A. After 7-day habituation, the mice were divided into four groups according to the sucrose preference baseline as detailed: 1) control mice treated with vehicle (Ctrl); 2) restrained mice treated with vehicle (Veh); 3) restrained mice treated with the traditional antidepressant fluoxetine (Flx); and 4) restrained mice treated with YL-0919 (YL). A 14-day treatment period is generally considered sub-chronic, which is longer than the minimum requirement for antidepressant action. To determine the astrocytic effects of YL-0919 in the CRS model, we first applied the OFT to assess motor ability. The results showed no significant difference in total distance across groups (Figures 1B, C right, F (3,36) = 0.5748, *p* = 0.6353). Notably, obvious depressive-like behaviors in mice were induced by CRS, including a reduced distance in the central zone in the OFT (Figures 1B, C left, *p* = 0.0004), a lower sucrose preference in the SPT (Figure 1D, *p* = 0.0080), and increased immobility in the TST (Figure 1E, *p* = 0.0124) and FST (Figure 1F, *p* < 0.0001). These results indicated that YL-0919 but not fluoxetine concomitant treatment significantly alleviated CRS-induced depression-like behaviors.

### YL-0919 alleviated the astrocyte deficits in the mPFC and HIP induced by CRS

**FIGURE 2:**
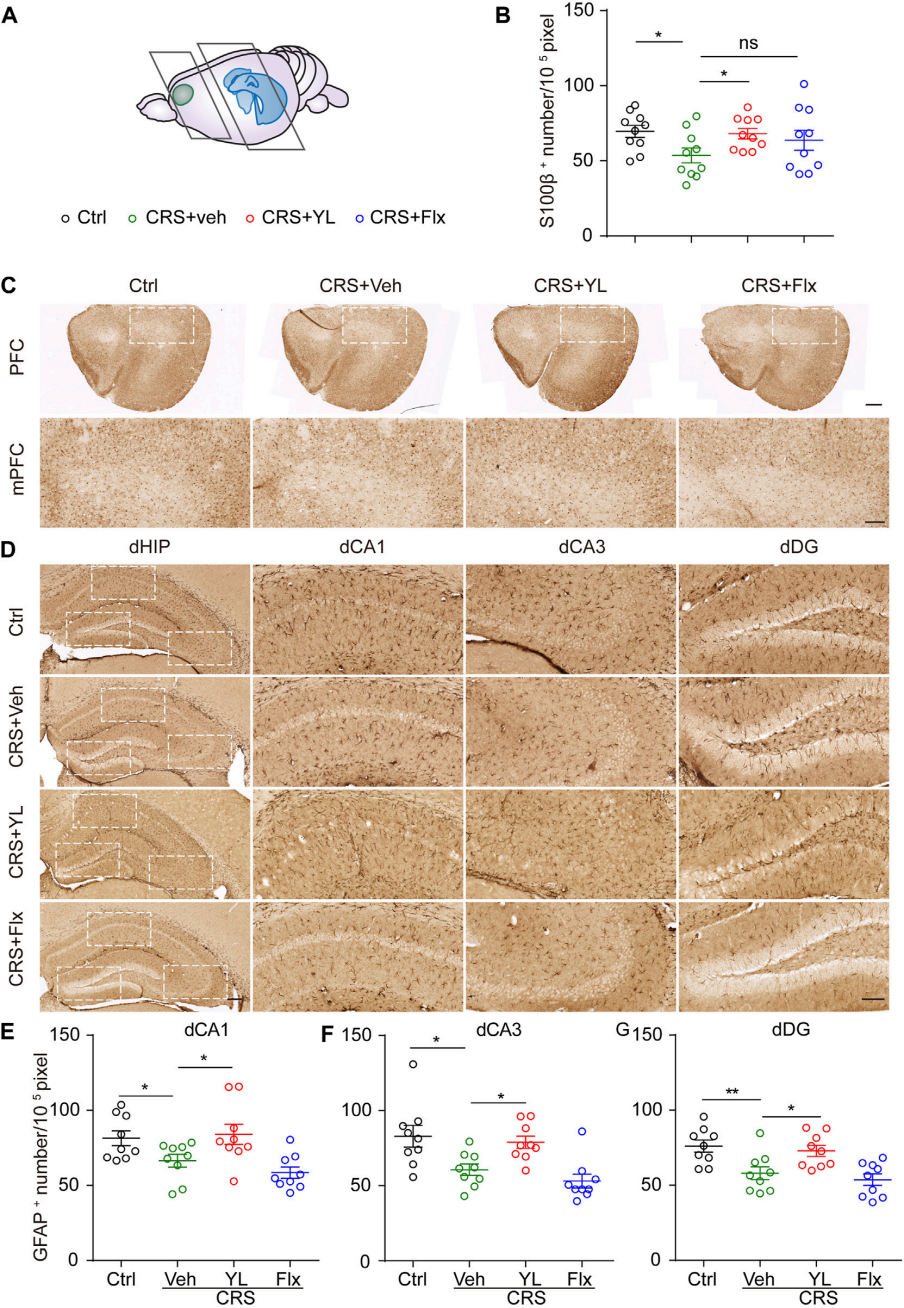
YL-0919 reverses astrocyte loss in mPFC and dHIP. **(A)** Schematic representation depicting coronal section areas of the mPFC and HIP. **(B)** Quantitative analysis of S100β^+^ cell numbers in the mPFC (F (3,36) = 2.184, *p* = 0.1069). **(C)** Typical images of S100β immunohistochemical staining in the PFC (upper panel, scale bar, 500 μm) and mPFC (lower panel, scale bar, 200 μm). Immunohistochemical staining of GFAP in the dorsal hippocampus subfields including dVA1 **(D)**, dCA3 **(F),** and dDG **(G)** (scale bar, 200 μm and 100 μm). Quantitative analysis of GFAP^+^ cell numbers in dCA1 **(E)** (F (3, 32) = 5.835, *p* = 0.0027), dCA3 **(F)** (F (3, 32) = 7.919, *p* = 0.0004), and dDG **(G)** (F (3, 32) = 7.671, *p* = 0.0005). Data are expressed as mean ± SEM, *n* = 10 in each group. For S100β^+^ and GFAP^+^ numbers in the vCA1 analysis, one-way ANOVA, followed by Fisher’s LSD, was used; otherwise, Bonferroni’s multiple comparisons test was applied. *p < 0.05; **p < 0.01.

As the mPFC and HIP are crucial brain areas for emotion disorders, we first assessed the number of astrocytes in the mPFC and HIP marked with S100β or GFAP, respectively. The number of S100β^+^ cells in the mPFC was significantly decreased after CRS, which was partially increased following treatment with YL-0919 but not fluoxetine (Figures 2B, C). Similarly, CRS significantly decreased the number of GFAP^+^ cells in the dorsal HIP (dHIP) subfields, which were partially restored only by YL-0919. YL-0919 treatment led to an almost complete preservation of GFAP^+^ astrocytes compared with the CRS group. In contrast, no significant difference was observed between the Flx and CRS groups (Figures 2D–G).

**FIGURE 3:**
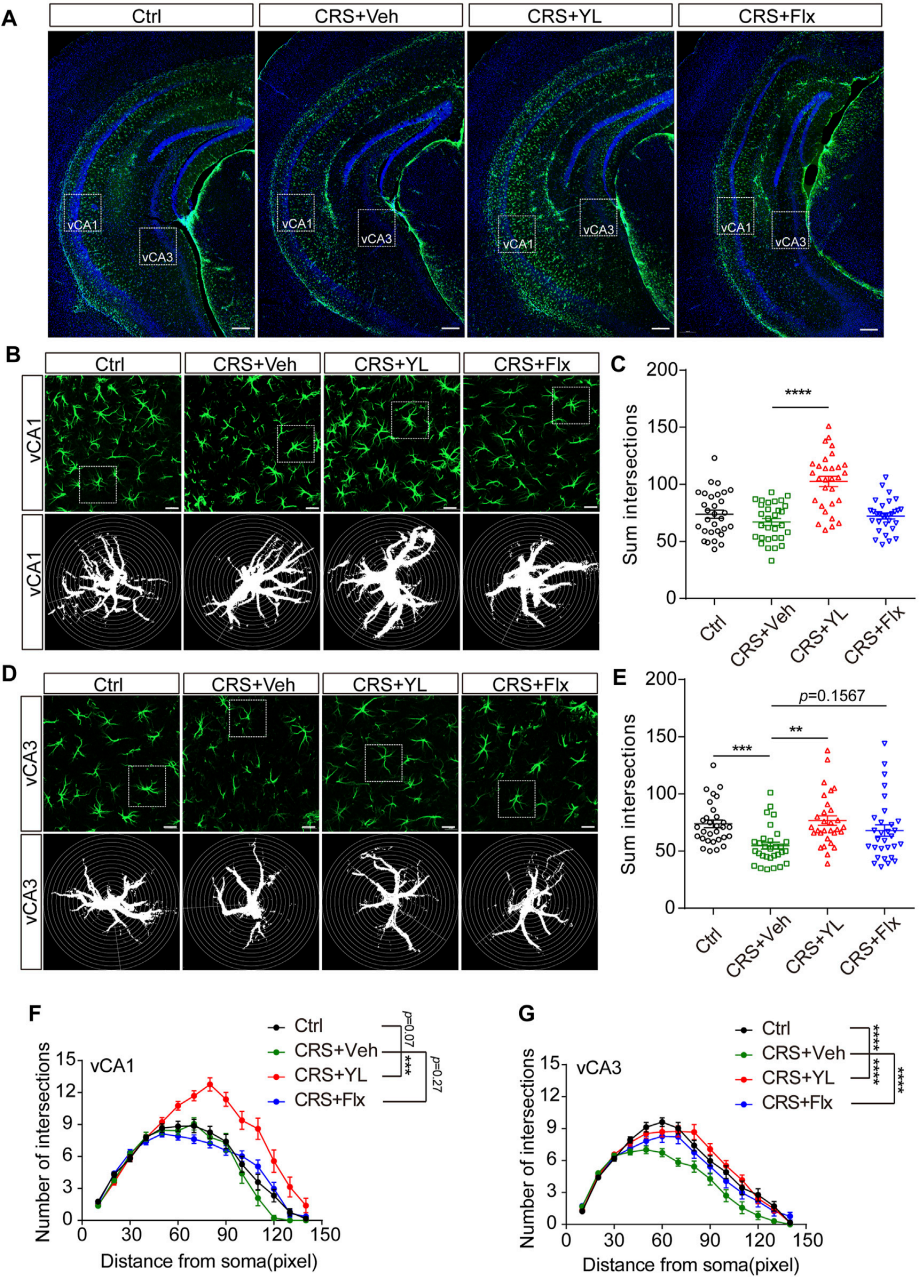
Effects of antidepressants on the morphology of astrocytes in the vHIP. **(A)** Immunofluorescent confocal images in the vHIP with astrocyte markers GFAP (green) and DAPI (blue) (scale bar: 200 µm). **(B)** Typical images of the vCA1 and **(D)** vCA3 shown at higher magnification (upper panel) and schematic representations of the Sholl analysis (lower panel, scale bar: 20 µm). Sum intersections of vCA1 **(C)** (F (3, 116) = 20.99, *p* < 0.0001) and vCA3 **(E)** astrocytes. Number of intersections in vCA1 **(F)** (F (3, 1624) = 69.96, *p* < 0.0001) and vCA3 **(G)** (F (3, 1624) = 35.19, *p* < 0.0001) astrocytes in each group. Data are mean ± SEM. *n* = 30 cells from five mice per group. For Sholl analysis, two-way ANOVA, followed by Bonferroni’s multiple comparisons test, was used. For sum intersection analysis, one-way ANOVA, followed by Tukey’s multiple comparisons test, was applied. *p < 0.05; **p < 0.01; ***p < 0.001; ****p < 0.0001.

Based on the aforementioned results, we further performed immunofluorescence staining to assess the morphology of astrocytes in the ventral HIP (vHIP). Astrocytes displayed abnormal morphology in the HIP after CRS (Figure 3A). The complexity of astrocytes processes was estimated by Sholl analysis, as previously described (Dinca et al., 2022). The CRS mice showed fewer intersections in CA1 astrocytes compared with the Ctrl group, which was more significant in processes located 60–70 pixels from the nucleus (Figures 3B, C, F). However, the reduction was more severe in vCA3 astrocyte processes located 50–60 pixels from the nucleus (Figures 3D, E, G). Interestingly, YL-0919 treatment reversed the reduction of astrocyte arborization in both vCA1 and vCA3, while fluoxetine showed partial improvement in vCA3 (Figures 3F, G). These results demonstrated that YL-0919 alleviated the CRS-induced astrocyte loss and atrophy in the mPFC and HIP.

### RNA-Seq analysis of YL0919 acting through astrocytes

**FIGURE 4:**
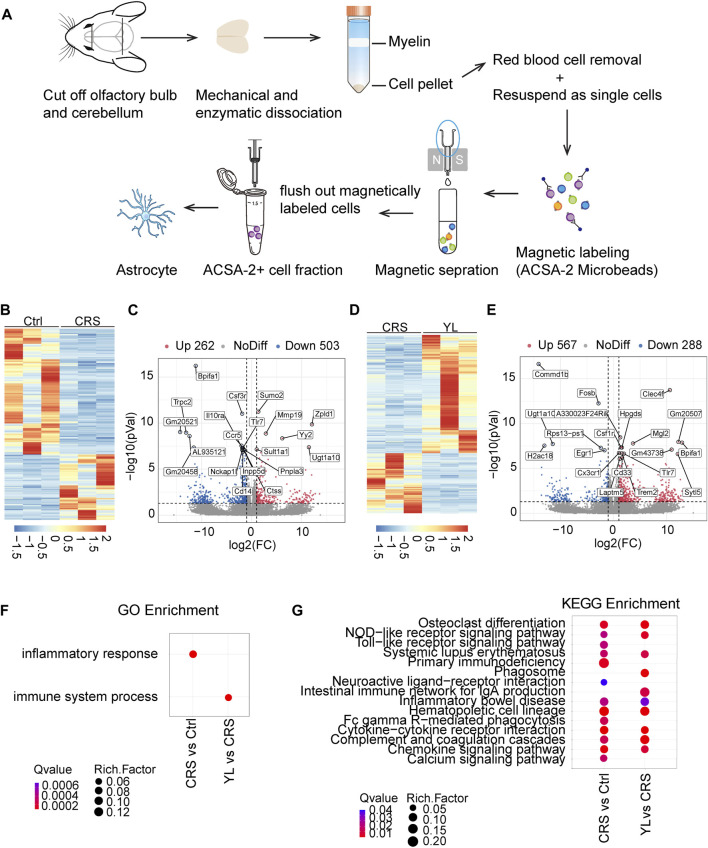
Gene expression profiling of astrocytes in CRS and YL-0919-treated mice revealed by RNA-Seq analysis. **(A)** Schemes depicting the column-based magnetic cell selection. **(B)** Heatmap and **(C)** volcano plot visualization of the gene profiling expression between the Ctrl and CRS groups. The heatmap **(D)** and volcano **(E)** show DEGs between the YL and CRS groups. Red: significantly upregulated genes; gray: genes with no significant differential expression; blue: significantly downregulated genes. The top 20 genes are shown (*p* < 0.05 and |log2FC| > 1). **(F)** Summary of gene ontology (GO) analysis of DEGs from the Ctrl, CRS, and YL groups. **(G)** Comprehensive comparison of significantly enriched pathways (Q < 0.05) of all comparison groups. The bubble colors and sizes, respectively, indicate the Q value and the number of differential genes of the pathway in the enrichment analysis. Q value > 0.05, the bubble is missing.

To gain insights into the underlying astrocytic mechanisms of YL-0919 treatment, we performed parametric RNA sequencing (RNA-Seq) analysis to capture transcriptome-wide alterations of brain astrocytes. To selectively isolate and purify astrocytes, we took advantage of the ACSA-2 antibody, following a recently described protocol for magnetic-assisted cell sorting (MACS) (Pan et al., 2022). Cells isolated from Ctrl, CRS, and YL mice were used for RNA-seq (Figure 4A). The gene expression profiles were first compared between CRS and Ctrl, which revealed a total of 548 significantly differential expression genes (DEGs), including 262 that were upregulated and 563 that were downregulated (*p* < 0.05 and |log2 (fold change)| ≥ 1, Figure 4B). The top 20 DEGs are displayed in Figure 4C. Compared to CRS mice, a total of 855 DEGs were identified after YL-0919 treatment, most of which were upregulated (567 vs. 288 that were downregulated) (*p* < 0.05 and |log2 (fold change)| ≥ 1, Figures 4D, E). To annotate the properties of DEGs in different biological functions, GO and KEGG analyses were performed to compare the enrichment differences among different groups. GO analysis showed that the biological process of inflammatory response was the most significantly enriched in the CRS group compared with the Ctrl group, while the molecular functions of the immune system process were most significantly enriched in the YL group compared with the CRS group (Figure 4F). We also detected altered genes involved in “primary immunodeficiency” and “hematopoietic cell lineage” in astrocytes of the CRS group (Figure 4G), as well as “osteoclast differentiation,” “cytokine–cytokine receptor interaction,” and “complement and coagulation cascades” in the YL group.

**FIGURE 5:**
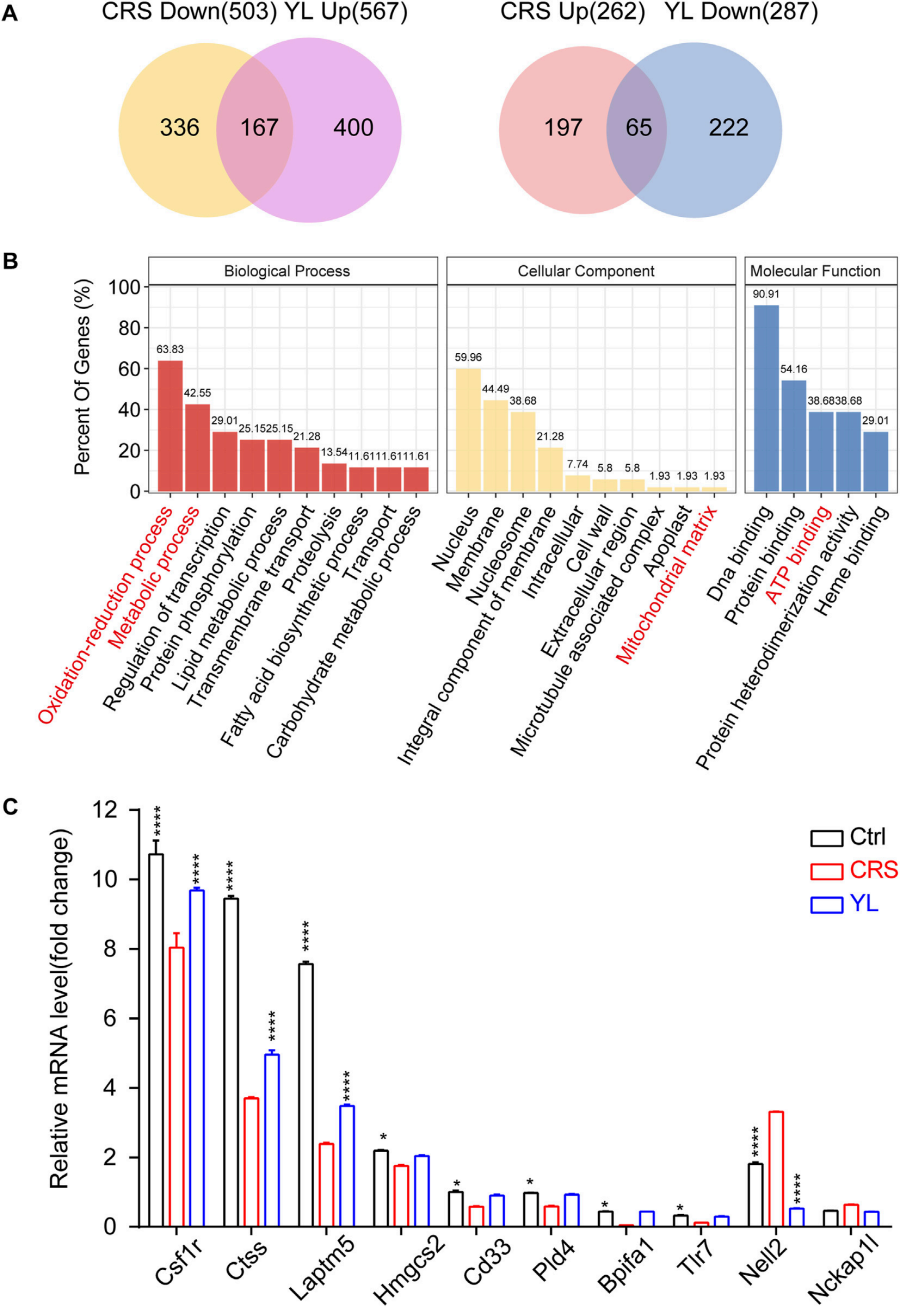
Venn screening and validation of oppositely changed DEGs. **(A)** Venn diagrams depicting the extent of overlap between genes downregulated (upregulated) in CRS mice and upregulated (downregulated) in YL-0919 treated mice. **(B)** Functional enrichment analysis of the overlapping genes indicating significantly enriched GO terms in the biological processes (red), cellular component (yellow), and molecular function (blue) categories. **(C)** qRT-PCR validation of the RNA-Seq data set with selected genes. Data are represented as mean ± SEM. One-way ANOVA, followed by Fisher’s LSD, was used. *n* = 3 in each group. Ctrl/YL vs. CRS. *p < 0.05; ****p < 0.0001.

To further narrow the scope of screening genes and identify more core changes, we performed Venn visualization of the two groups of differential genes, which revealed that 65 genes were upregulated in the CRS model and downregulated after the administration of YL-0919, while 167 genes were downregulated in the CRS model and upregulated after the administration of YL-0919 (Figure 5A; Supplementary Figure S3). In addition, gene function enrichment analysis on these selected genes showed significant enrichment of metabolic processes, ATP binding, and mitochondrial matrix (Figure 5B). We then chose a total of 10 DEGs for qRT-PCR analysis, which confirmed the reliability of the RNA-Seq findings (Figure 5C). Taken together, these results indicated the effects of YL-0919 on mitochondrial function in astrocytes which leads to faster relief from a depressive state.

### YL0919 improves mitochondrial OXPHOS in astrocytes

**FIGURE 6:**
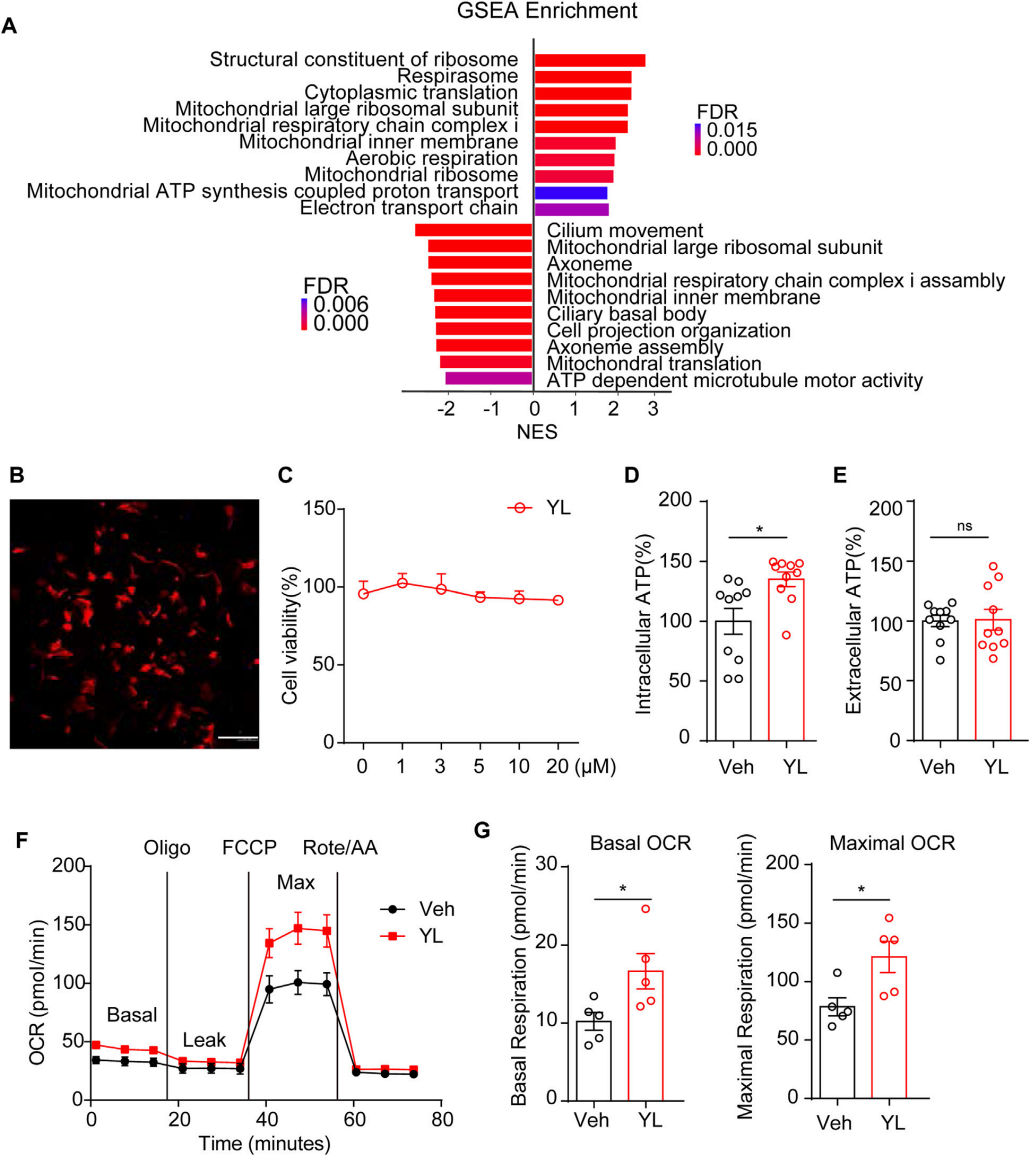
Impact of YL-0919 on mitochondria function in primary astrocytes. **(A)** Summary of GSEA performed on the FDR-ranked gene list. **(B)** Representative image of cultured primary astrocytes *in vitro*. GFAP (red) and DAPI (blue); scale bar, 200 μm. **(C)** Astrocyte cell viability was expressed as a percentage of the control, which was set to 100%. Cells were treated with different concentrations of YL-0919 for 24 h. Data are expressed as means ± SEM and analyzed by one-way ANOVA, F (5, 24) = 1.046, *p* = 0.4139, and *n* = 10. Intracellular **(D)** and extracellular **(E)** ATP levels of Veh and YL-0919-treated astrocytes. **(F)** OCR measurements of Veh and YL-0919-treated astrocytes. Statistics on basic OCR **(G left)** and maximum OCR **(G right)**. Data are expressed as means ± SEM and analyzed by two-tailed Student’s t-test. *n* = 10 in ATP. *n* = 5 in OCR. *p < 0.05.

Finally, to make up for the lack of effective information from minor genes by traditional enrichment analysis, we further adopted GSEA analysis, which can more comprehensively explain the regulatory effects of a certain functional unit and complement traditional enrichment analysis. The GSEA results suggested changes in mitochondria-related functions including after CRS treatment and YL-0919 administration (Figure 6A). Therefore, we designed experiments to verify the effects of YL-0919 on the energy metabolism of astrocytes. First, we treated isolated primary astrocytes (Figure 6B) with different concentrations of YL-0919 (0–20 μM). Data from the CCK-8 test (Figure 6C) indicated no significant change in cell viability after 24 h of incubation, following YL-0919 treatment at any dosage (F (5, 24) = 1.046, *p* = 0.4139). ATP levels measured after 24 h of incubation with Veh or YL-0919 (5 μM) showed that YL-0919 significantly increased intracellular ATP levels (Figure 6D, *p* = 0.0111) but not extracellular ATP production (Figure 6E, *p* = 0.9160) in astrocytes, while the production of cellular ATP was mainly due to mitochondrial oxidative phosphorylation and glycolysis. Therefore, we examined the effect of YL-0919 on the oxygen consumption rate (OCR) of astrocytes. The results showed that YL-0919 increased the oxidative respiration capacity of astrocytes (Figure 6F), which manifested as increased basal oxygen consumption (Figure 6G left, *p* = 0.0361) and maximal oxygen consumption (Figure 6G right, *p* = 0.0248), indicating that YL-0919 increased the oxidative phosphorylation capacity of astrocytes, thus strongly supporting the role of YL-0919 in the energetic metabolism of astrocytes.

## Discussion

Previous studies demonstrated the marked antidepressant effects of YL0919 in different animal models (Zhang et al., 2017; Ran Y. et al., 2018; Ran Y. H. et al., 2018; Sun et al., 2019; Yin et al., 2020; Zhang et al., 2022); however, the astrocyte-specific mechanism remains unclear. Therefore, the present study first demonstrated that YL-0919 significantly ameliorated CRS-induced depression-like behaviors in mice more rapidly than fluoxetine. Moreover, YL-0919 increased astrocyte numbers in the mPFC and HIP and reversed the declining morphological complexity of astrocytes, especially in vCA3. More importantly, RNA-Seq revealed that the transcriptome alterations in astrocytes induced by CRS mainly affected inflammatory and immune response and that the expression levels returned to normal after YL-0919 treatment. GO and KEGG analysis showed that the aforementioned genes were mainly enriched in mitochondria-related functions. Further experiments *in vitro* demonstrated that YL-0919 significantly promoted astrocyte ATP synthesis and improved cellular oxidative phosphorylation, which verified the accuracy of the transcriptome sequencing results. Our data suggest that astrocytes play an important role in stress-induced depression and that the faster-onset antidepressant mechanism of YL-0919 may work by altering astrocyte plasticity and maintaining mitochondrial metabolic function.

Substantial evidence has identified that structural and functional abnormalities of astrocytes play a crucial role in depression progression (Machado-Santos et al., 2021). The long-term administration of fluoxetine can rescue the decline in astrocyte number (Czeh et al., 2006). S100β acts as a neurotrophic factor to stimulate the local proliferation of astrocytes and fluoxetine can improve S100β levels in the HIP and CSF (Baudry et al., 2010). GFAP is a key skeletal protein of mature astrocytes and is often used as a marker of reactive astrocytes (Huang et al., 2022). Mice in depression models such as chronic unpredictable mild stress (CUMS) showed decreased GFAP^+^ cell density, while riluzole reversed decreased GFPA mRNA expression and promoted astrocyte metabolism in CUMS mice (Banasr et al., 2010). Hence, we examined astrocyte numbers *via* S100β or GFAP expression. Our data showed that YL-0919 significantly increased S100β^+^ and GFAP^+^ cell density, respectively, in the mPFC and various subfields in the HIP of CRS mice, suggesting that astrocytes are key mediators of the faster-onset antidepressant effect of YL-0919 (Figures 1, 2).

Cumulative studies have indicated that the HIP is sensitive to MDD progression. A reduction in HIP volume is inversely correlated with the frequency and untreated duration of MDD (Frodl et al., 2008) but can be partially rescued by antidepressant therapy (Schermuly et al., 2011). Notably, the HIP has functional heterogeneity along its dorsal-ventral axis. The dHIP mainly performs cognitive functions, such as learning and memory, while the vHIP is more associated with stress and mood (Fanselow and Dong, 2010). Recent studies have found that stress can result in decreased astrocyte numbers in the dHIP of mice and induce atrophy and dysfunction (Murphy-Royal et al., 2019), consistent with our results (Figure 2). However, few studies have reported on the changes in astrocytes in the vHIP under depression. Moreover, reduced HIP was found only in the CA2-4 regions in patients newly diagnosed with MDD and other subregions, including the DG, subiculum, and CA, in patients with recurrent MDD (Roddy et al., 2019). In our study, YL-0919 was found for the first time to significantly reverse the CRS-induced astrocyte atrophy in the vCA3 region, while the 14-day fluoxetine treatment showed no recovery of the depressive-like behaviors and mPFC and HIP astrocytic loss. However, fluoxetine can increase the morphological complexity of astrocytes in the vCA3 region (Figure 3). In conclusion, the pathological changes of depression and the effects of the drug first involve CA2-4, before gradually extending to other subregions of the HIP. The enhancement of astrocytic process complexity in vCA3 and the subsequent recovery of morphology and number in the whole HIP are the pivotal mechanisms of the faster onset of antidepressant effects by YL-0919.

To identify potential key molecules of MDD, large-scale unbiased screenings are usually performed using omics (Wingo et al., 2021). With advances in genomic approaches and genetic manipulation, we can explore genomic changes in specific cell types (Burda et al., 2022). TRAP-seq results showed that chronic stress reduced the levels of genes associated with astrocyte plasticity, including Rho GTPase and genes involved in growth factor signaling transduction and transcriptional regulation, but increased genes associated with extracellular matrix formation (Simard et al., 2018). Therefore, we applied RNA-Seq to comprehensively and specifically reveal changes in gene expression after the development of depression or antidepressant treatment. Enrichment analysis showed that the expression levels of CRS vs Ctrl genes were mainly downregulated and enriched in immune process, biological process of signal transduction, cellular components of extracellular matrix and membrane, and molecular function of protein binding, among others (Figure 4) (Supplementary Figure S1). However, the comparison of differential genes in YL vs CRS showed mainly upregulation and enrichment, especially in the cellular components of cell projection, synapse, cytoplasmic vesicle, and molecular function of ATP binding (Figure 4) (Supplementary Figure S2). Our findings not only shed light on the changes in gene expression profiles in astrocytes after CRS but also demonstrate the novel astrocytic mechanism of the antidepressant effects of YL-0919 at the transcriptome level.

Mitochondria are required to establish and maintain immune cell phenotypes and functions (Mills et al., 2017). Recently, a growing body of evidence suggests that mitochondrial dysfunction may play a crucial role in MDD pathogenesis (Simon et al., 2021). Mitochondrial perturbation and release of mitochondrial components promoted cytokine production and neuroinflammation in depression models (Allen et al., 2021); conversely, pro-inflammatory cytokines may affect mitochondrial function such as oxidative phosphorylation and the production of ATP and ROS (reactive oxygen species), which exacerbate inflammation. Mitochondria can rapidly transform from catabolic organelles, which mainly produce ATP, to anabolic organelles, which also synthesize macromolecules, to meet the metabolic needs of various immune cells (Breda et al., 2019). Moreover, mitochondria can activate multiple signaling pathways, including changing the AMP/ATP ratio, release of ROS and TCA circulating metabolites, and localization of immunomodulatory proteins on the mitochondrial outer membrane (Voss et al., 2021). GSEA analysis in the present study revealed the enrichment of genes related to the mitochondrial function of astrocytes. The results of the Seahorse extracellular flux assay verified the difference in the energy activity of astrocytes after YL-0919 treatment (Figure 5), further validating that YL-0919 has faster-onset antidepressant effects by improving the mitochondrial function of astrocytes.

Astrocytes have extensive spatial heterogeneity (Batiuk et al., 2020; Burda et al., 2022). The results of the whole-brain astrocyte RNA-Seq revealed the potential mechanisms which occurred in but were not specific to mPFC and HIP, providing the foundation for subsequent research. However, due to several limitations, the present study did not explore the genetic and functional changes of astrocytes in different subregions across different subsets in greater detail. Since drugs do not only act on a single type of cells or molecules, future studies will further explore the interactions between astrocytes and other cells and search for potential targets of antidepressants *via* multiple mechanisms.

## Conclusion

We have, for the first time, revealed a new astrocyte-specific mechanism for the antidepressant YL-0919, in which YL-0919 improves CRS-induced number and morphological deficits in astrocytes, partially reverses changes in the astrocyte transcriptome, and promotes ATP production and mitochondrial oxidative phosphorylation. Our results provide novel ideas for further elucidating the pathogenesis of depression and discovering promising targets of antidepressants based on astrocytes.

## Data Availability

The original contributions presented in the study are publicly available. This data can be found here: [https://www.ncbi.nlm.nih.gov/PRJNA944268].
