# Supplementary material for: Astrocytes underlie a faster-onset antidepressant effect of hypidone hydrochloride (YL-0919)
Source: Front Pharmacol. 2023 Mar 29;14:1175938. doi: 10.3389/fphar.2023.1175938 (PMC10090319; doi:10.3389/fphar.2023.1175938)
Supplement: Supplementary file 1 [file DataSheet1.PDF]

Supplementary Material

Astrocytes underlie a faster-onset antidepressant effect of hypidone hydrochloride (YL-0919)

Jinfeng Li[1], Wenyu Hu[1,2], Haixia Chang[1,3], Jinhao Bao[1], Xiangxi Kong[1,4*], Hui Ma[1*], Yun-Feng Li [1*]

[1] Beijing Institute of Basic Medical Sciences, Beijing, 100850, China.

[2] Institute of Neuroscience, Hengyang Medical College, University of South China, Hengyang, 421001, China.

[3] College of Pharmacy, Shandong First Medical University & Shandong Academy of Medical Sciences, Taian, 271016, China.

[4] Jiangsu Province Key Laboratory of Anesthesiology, Jiangsu Province Key Laboratory of Anesthesia and Analgesia Application, NMPA Key Laboratory for Research and Evaluation of Narcotic and Psychotropic Drugs, Xuzhou Medical University, Xuzhou 221004, China.

[5] Beijing Institute of Pharmacology and Toxicology, State Key Laboratory of Toxicology and Medical Countermeasures, Beijing Key Laboratory of Neuropsychopharmacology, Beijing, 100850, China.

*** Correspondence:**
Yunfeng Li, lyf619@aliyun.com

Hui Ma, mahui_bjmu@163.com

Xiangxi Kong, kongxiangxi1989@163.com

Primer	Forward sequence	Reverse sequence
Bpifa 1	TGCCTTTGGCTGTAAGCCC	AGAATTGCCTCCTCCAGACTTTA
Nell2	ACGTTCTGCGTGATCCTCG	CGTCAATCTGTAGGGAGGGGT
Ctss	CCATTGGGATCTCTGGAAGAAAA	TCATGCCCACTTGGTAGGTAT
Tlr7	ATGTGGACACGGAAGAGACAA	GGTAAGGGTAAGATTGGTGGTG
Cd33	CCGCTGTTCTTGCTGTGTG	AAGTGAGCTTAATGGAGGGGTA
Laptm5	GATGCCGTACCTCAGGATGG	CTCCCGGTTCTTGACCACG
Pld4	ACCCAGAGATGCGGATACCA	CCCCTTGCCACAGGAACAA
Nckap1l	TGTCCGAAATAGCACGCAACA	ATCCCGAAATTCCATGACATCC
Cd37	GCCCAAGAGAGTTGCCTCAG	GGCCGCCTAGTACAAAGAAGAA
Hgcs2	GAAGAGAGCGATGCAGGAAAC	GTCCACATATTGGGCTGGAAA
β-actin	GGCTGTATTCCCCTCCATCG	CCAGTTGGTAACAATGCCATGT

Supplementary Table 1. The primer sequence used in this study. All primer sequence were generated from “PrimerBank” and synthesized by Sangon Biotech Co. (Shanghai, China)

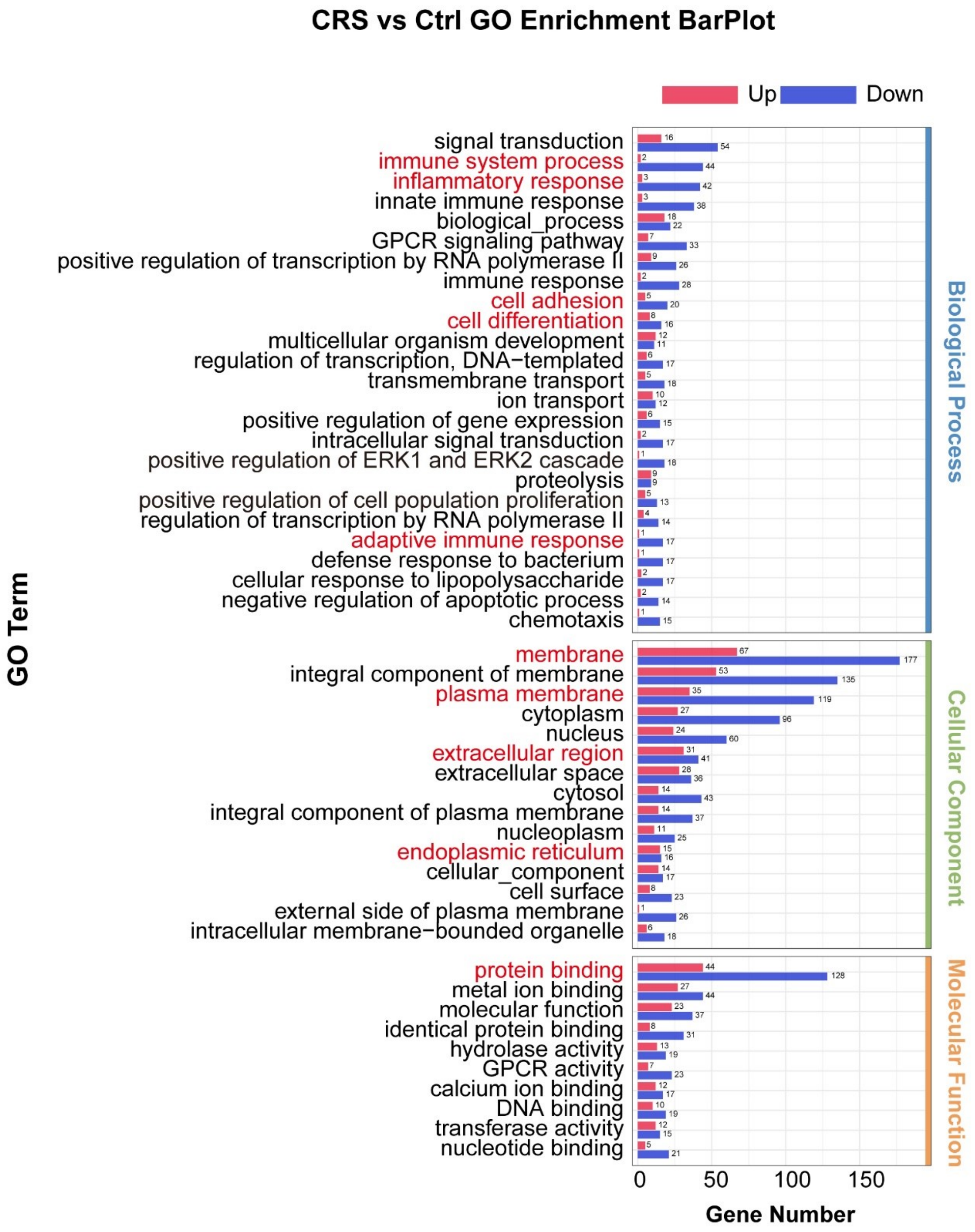

Supplementary Figure 1. GO analysis of DEGs from Ctrl and CRS group in biological process, cellular component and molecular function.

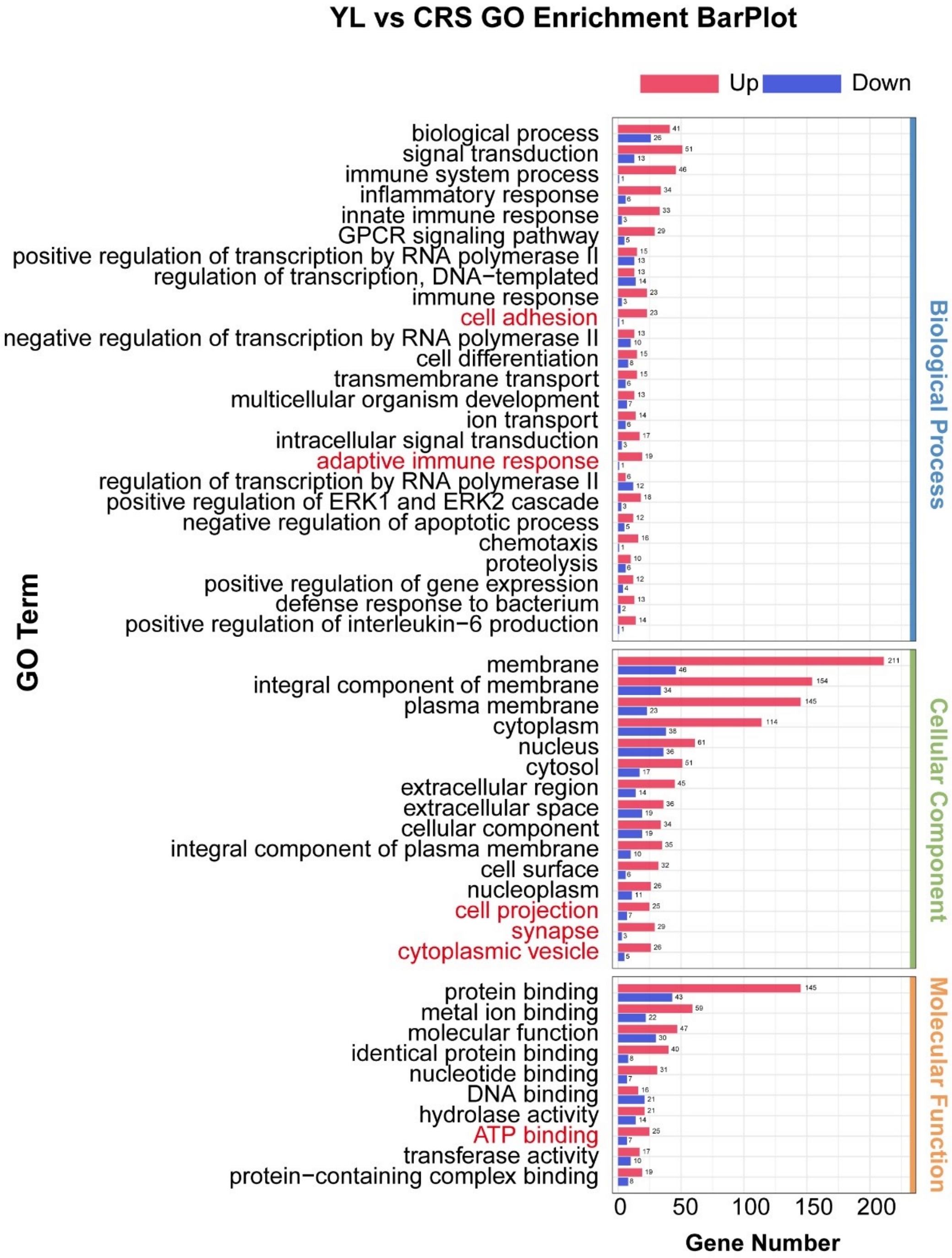

Supplementary Figure 2. GO analysis of DEGs from CRS and YL group in biological process, cellular component and molecular function.

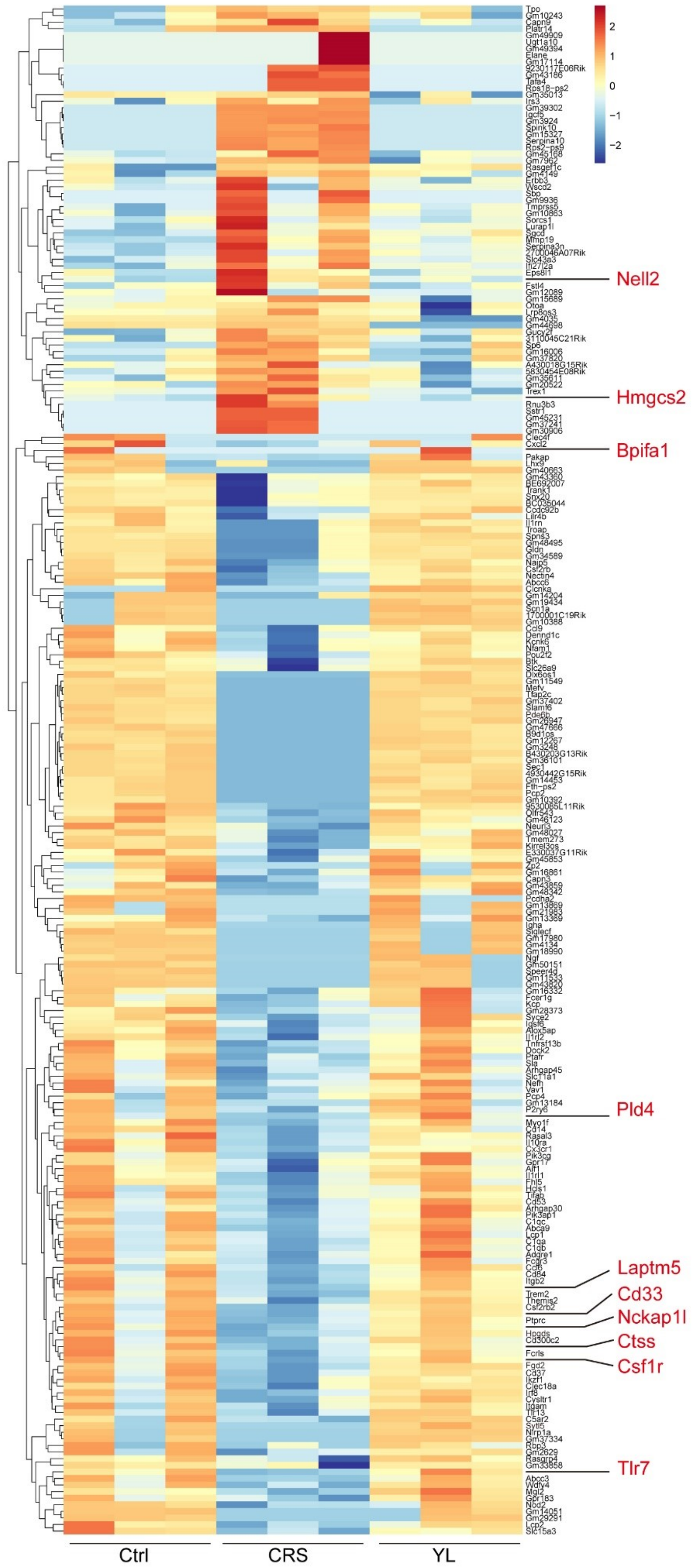
2
1
0
−1
−2
Nell2
Hmgcs2
Bpifa1
Pld4
Laptm5
Cd33
Nckap1l
Ctss
Csf1r
Tlr7
Ctrl
CRS
YL

Supplementary Figure 3. Heatmap visulization of DEGs in each groups screened through venn diagram.
